# Risk Factors Influencing Efficacy and Prognosis Evaluation of Neoadjuvant Systemic Therapy in Triple‐Positive Breast Cancer

**DOI:** 10.1002/kjm2.70228

**Published:** 2026-05-07

**Authors:** Lin‐Rui Miao, Hao Liu, Hai‐Ying Yu, Jian Chen, Ming‐Yu Yang, Ya‐Lan Zhang, Gang Tu, Ling‐Feng Tang

**Affiliations:** ^1^ Department of Breast and Thyroid Surgery, Chongqing Key Laboratory of Molecular Oncology and Epigenetics The First Affiliated Hospital of Chongqing Medical University Chongqing China; ^2^ Department of Oncology Chongqing Hygeia Hospital Chongqing China

**Keywords:** neoadjuvant systemic therapy, nomogram, prognosis, triple‐positive breast cancer

## Abstract

To identify the determinants of the response to neoadjuvant systemic therapy (NST) in triple‐positive breast cancer (TPBC), we retrospectively enrolled 520 patients with human epidermal growth factor receptor 2 (HER2)‐positive breast cancer who were treated at The First Affiliated Hospital of Chongqing Medical University between January 2015 and December 2021. The cohort comprised 299 cases of TPBC and 221 cases of hormone receptor (HR)‐negative/HER2‐positive breast cancer (HPBC). A comparative analysis revealed that the pathological complete response (pCR) rate was significantly lower in the TPBC group than in the HPBC group (30.1% vs. 50.2%; *p* < 0.001); however, this differential response did not translate into a significant difference in long‐term survival. Within the TPBC cohort, pCR was identified as an independent prognostic factor for prolonged disease‐free survival (DFS) (*p* = 0.014). Multivariate analysis further revealed that the NST regimen (*p* = 0.001), estrogen receptor (ER) status (*p* = 0.024), and Ki67 index (*p* = 0.018) were independent predictors of pCR. A nomogram incorporating these factors was developed using R software to estimate the individual probability of pCR in TPBC. The model was externally validated in an independent cohort of 143 TPBC patients treated between January 2022 and December 2024, and the results demonstrated robust predictive performance and good calibration. This model serves as a tool for early risk stratification in TPBC, thereby facilitating personalized treatment strategies and risk‐adapted surveillance to improve patient outcomes.

**Abbreviations**
AR: androgen receptor
CR: complete response
DFS: disease‐free survival
ER: estrogen receptor
HER2: human epidermal growth factor receptor 2
HPBC: HR‐negative/HER2‐positive breast cancer
HR: hormone receptor
IHC: immunohistochemical
MLR: monocyte‐to‐lymphocyte ratio
NLR: neutrophil‐lymphocyte ratio
NST: neoadjuvant systemic therapy
pCR: pathological complete response
PD: progression disease
PR: progesterone receptor
PR^#^
: partial response
ROC curve: receiver operating characteristic curve
SD: stable disease
TNBC: triple‐negative breast cancer
TPBC: triple‐positive breast cancer

## 1 Introduction

Globally, breast cancer poses a major threat to women's health due to its high incidence. In China, the incidence rate has also exhibited a continuous upward trend [1]. International consensus classifies breast cancer into several subtypes, which are primarily stratified by the status of hormone receptor (HR) and human epidermal growth factor receptor 2 (HER2) expression [2]. Each subtype has heterogeneous clinicopathological characteristics, which govern variations in treatment efficacy and prognosis.

Triple‐positive breast cancer (TPBC), defined by the coexpression of estrogen receptor (ER), progesterone receptor (PR), and HER2, represents a unique entity, accounting for approximately 8%–10% of all breast cancer cases [3]. The management of TPBC extends beyond radical mastectomy to encompass neoadjuvant systemic therapy (NST) and postoperative adjuvant therapy. The standard regimen integrates chemotherapy with dual HER2 blockade and endocrine therapy [4].

NST facilitates tumor downstaging, enhances the feasibility of breast‐conserving surgery, and informs subsequent adjuvant treatment strategies based on the pathological response. NST is indicated for patients with locally advanced tumors, a high tumor burden, or a desire for breast conservation, as it can improve both survival and quality of life. However, evidence suggests that intracellular crosstalk between the ER and HER2 signaling pathways constitutes a key mechanism underlying therapeutic resistance and disease progression [5]. This mechanism regulates tumor cell proliferation and drug responsiveness at multiple levels, including cell cycle regulation, signal transduction, direct cytoplasmic interactions, and the tumor microenvironment [6, 7].

Whether ER‐HER2 crosstalk affects NST efficacy in TPBC remains a critical clinical question. To address this, we leveraged real‐world clinical data to identify the clinicopathological determinants of the NST response and long‐term survival in TPBC, thereby informing strategies to overcome therapeutic resistance.

## 2 Methods

### 2.1 Population

This retrospective analysis was conducted on clinicopathological data from patients managed at the Department of Breast and Thyroid Surgery, the First Affiliated Hospital of Chongqing Medical University, between January 2015 and December 2021. The inclusion criteria were as follows: (1) age ≥ 18 years; (2) invasive breast cancer diagnosed by core needle biopsy or vacuum‐assisted biopsy; (3) completion of NST, subsequent surgery, and standard adjuvant therapy at our institution; and (4) complete clinical and pathological records. The exclusion criteria included (1) male sex, (2) bilateral breast cancer, (3) inflammatory breast cancer, (4) other concurrent primary malignant tumors, or (5) incomplete medical records.

**FIGURE 1:**
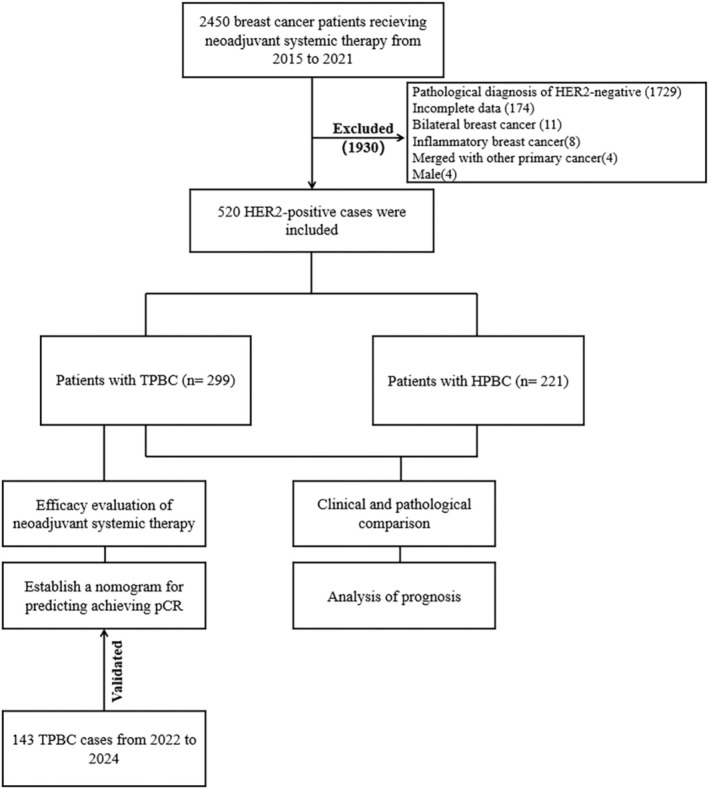
Study flowchart. Schematic showing patient selection, exclusion criteria, and group allocation in this retrospective cohort study.

Based on these criteria, a total of 520 patients with HER2‐positive breast cancer were included, comprising 299 patients with TPBC and 221 patients with HR‐negative/HER2‐positive breast cancer (HPBC). Clinicopathological characteristics were systematically analyzed, and a predictive model for NST efficacy was constructed. An additional validation cohort of 143 TPBC patients treated between January 2022 and December 2024 was subsequently utilized to assess the performance of the predictive model (Figure 1).

This study was approved by the Institutional Ethics Committee of the First Affiliated Hospital of Chongqing Medical University (no. 2022‐202). Because this was a retrospective analysis utilizing anonymized clinical data, the requirement for informed consent was waived.

### 2.2 Clinicopathologic Characteristics

Patient data were retrospectively collected from the hospital's internal system and supplemented by telephone follow‐ups. The variables included age, menstrual status, complete blood cell counts, sex hormone levels, immunohistochemical profiles (ER, PR, Ki67, HER2, P53, E‐cadherin, and androgen receptor [AR] status), histological grade, TNM stage, NST regimen, surgical procedure, radiotherapy records, recurrence, and survival outcomes.

Histological grading was assessed according to the Nottingham Grading System based on three aspects: tubule formation, nuclear pleomorphism, and mitotic count. The final scores were categorized as follows: 3–5 points for Grade I (well differentiated), 6–7 points for Grade II (moderately differentiated), and 8–9 points for Grade III (poorly differentiated) [8]. HR positivity was defined as ≥ 1% of tumor cells exhibiting nuclear immunostaining. HER2 positivity was defined as an IHC 3+ or an IHC 2+ with confirmation of gene amplification by fluorescence in situ hybridization (FISH) confirmation. The Ki67 index was calculated as the percentage of Ki67‐positive tumor nuclei among all viable tumor cells. T staging was determined through clinical examination and breast imaging (enhanced MRI or ultrasound). N and M staging were assessed using appropriate imaging modalities, with pathological confirmation when indicated.

### 2.3 Treatment

NST refers to the initial systemic treatment administered to patients with breast cancer prior to planned definitive surgery. The primary objectives of NST are to downstage the tumor, enhance surgical outcomes, and provide an in vivo assessment of the treatment response to guide subsequent therapeutic decisions [9].

All patients underwent rigorous eligibility assessment prior to initiating NST. Indications for NST included (1) locally advanced breast cancer; (2) tumors > 2 cm or axillary lymph node involvement in triple‐negative breast cancer (TNBC) or HER2‐positive subtypes; and (3) initial tumor size precluding breast‐conserving surgery in patients with a strong desire for breast preservation [10].

**FIGURE 2:**
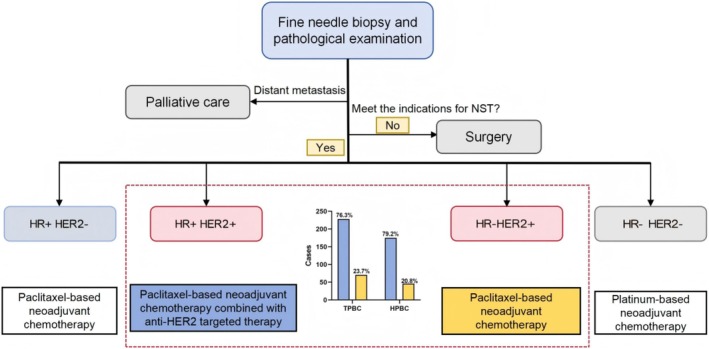
Prior to initiating NST, all patients underwent rigorous eligibility assessment. Treatment regimens were selected based on molecular subtype and individual patient characteristics. The majority of patients in both the HPBC and TPBC groups received paclitaxel‐based neoadjuvant chemotherapy combined with anti‐HER2 targeted therapy. However, approximately 20% of patients in each group received neoadjuvant chemotherapy alone due to insurance restrictions or financial constraints.

The selection of NST was tailored according to molecular subtype, anticipated drug sensitivity, and individual patient factors, primarily comprising chemotherapy alone or in combination with anti‐HER2 targeted agents. Prior to initiating neoadjuvant anti‐HER2 therapy, HER2 positivity in all patients was confirmed by IHC. The standard dual‐blockade regimen of intravenous trastuzumab and pertuzumab was administered throughout the neoadjuvant period. The selection of NST regimens was mostly guided by molecular subtype, as detailed in Figure 2.

The specific options included the following: (1) paclitaxel‐based neoadjuvant chemotherapy: TEC/TAC: docetaxel 75 mg/m^2^ or paclitaxel‐albumin 260 mg/m^2^ on Day 1; epirubicin 75 mg/m^2^ or pirarubicin 50 mg/m^2^ on Day 1; cyclophosphamide 500 mg/m^2^ on Day 1; 21 days per cycle, for a total of six cycles. (2) Paclitaxel‐based neoadjuvant chemotherapy combined with anti‐HER2 targeted therapy: TCbHP: docetaxel 75 mg/m^2^ or paclitaxel‐albumin 260 mg/m^2^ on Day 1; carboplatin AUC = 6 on Day 1; trastuzumab 6 mg/kg (first dose 8 mg/kg) on Day 1; pertuzumab 420 mg (first dose 840 mg) on Day 1; 21 days per cycle, for a total of six cycles.

Patients were evaluated for standardized efficacy after every two cycles of NST, with evaluation methods maintained consistently before and after treatment. Responses were classified as complete response (CR), partial response (PR^#^), stable disease (SD), or progressive disease (PD) [11]. The treatment strategy was adapted based on the response assessment. Disease progression or stability (PD/SD) prompted reconsideration of the management plan, with recommendations for either early surgery for operable cases or an immediate switch to an alternative therapeutic regimen.

### 2.4 Surgery

Following NST completion, a comprehensive assessment is needed to determine the optimal timing and approach for subsequent surgery. The choice should be individualized. Candidates for breast conservation surgery must meet the following criteria: (1) a favorable tumor‐to‐breast volume ratio to ensure acceptable cosmetic outcomes; (2) the feasibility of complete tumor excision with negative margins; and (3) suitability for postoperative radiotherapy [12]. In this study, axillary lymph node dissection was routinely performed after NST.

The efficacy of NST was evaluated primarily by pathological response, with pathological complete response (pCR) defined as the absence of invasive tumor cells in the primary breast lesion and regional lymph nodes.

### 2.5 Statistical Methods

Statistical analyses were performed using R (version 4.0.5) and SPSS (version 26.0). The chi‐square test or Fisher's exact test was used to compare categorical variables, as appropriate. Independent risk factors were identified through univariate and multivariate logistic regression analyses. The discriminative ability of the predictive model was assessed by the area under the receiver operating characteristic (ROC) curve, while consistency was evaluated by a calibration plot. A *p* value < 0.05 was considered to indicate statistical significance.

## 3 Results

### 3.1 Significantly Lower pCR Rate in TPBC Despite Shared Clinicopathological Features With Those in HPBC

The study cohort comprised 520 eligible patients and was categorized into a TPBC group (*n* = 299) and an HPBC group (*n* = 221). An initial analysis comparing the clinicopathological features between these groups is presented in Table 1.

**TABLE 1:** Baseline clinicopathological characteristics of TPBC and HPBC.

Characteristic		Total (*n* = 520)	TPBC (*n* = 299)	HPBC (*n* = 221)	*p*
Age (years)	≤ 45	155 (29.8%)	113 (37.8%)	42 (19.0%)	< 0.001*
	> 45	365 (70.2%)	186 (62.2%)	179 (81.0%)	
Menopausal status	Premenopausal	277 (53.3%)	189 (63.2%)	88 (39.8%)	< 0.001*
	Postmenopausal	243 (46.7%)	110 (36.8%)	133 (60.2%)	
NLR index	≤ 1.96	241 (46.3%)	136 (45.5%)	105 (47.5%)	0.647
	> 1.96	279 (53.7%)	163 (54.5%)	116 (52.5%)	
MLR index	≤ 0.20	200 (38.5%)	130 (43.5%)	70 (31.7%)	0.006*
	> 0.20	320 (61.5%)	169 (56.5%)	151 (68.3%)	
E2	≤ 61.5 pg/mL	388 (74.6%)	229 (76.6%)	159 (71.9%)	0.229
	> 61.5 pg/mL	132 (25.4%)	70 (23.4%)	62 (28.1%)	
T staging	I	62 (11.9%)	38 (12.7%)	24 (10.9%)	0.701
	II	369 (71.0%)	208 (69.6%)	161 (72.9%)	
	III/IV	89 (17.1%)	53 (17.7%)	36 (16.3%)	
N staging	N0	148 (28.5%)	103 (34.4%)	45 (20.4%)	0.002*
	N1	193 (37.1%)	100 (33.4%)	93 (42.1%)	
	N2/N3	179 (34.4%)	96 (32.1%)	83 (37.6%)	
Histological grading	Grade 1/2	392 (75.4%)	157 (71.0%)	235 (78.6%)	0.048*
	Grade 3	128 (24.6%)	64 (29.0%)	64 (21.6%)	
ER status (%)	[1, 20)		70 (23.4%)		
	[20, 50)		109 (36.5%)		
	≥ 50		120 (40.1%)		
PR status (%)	[1, 50)		193 (64.5%)		
	≥ 50		106 (35.5%)		
P53 status	Negative	151 (29.0%)	84 (28.1%)	67 (30.3%)	0.581
	Positive	369 (71.0%)	215 (71.9%)	154 (69.7%)	
Ki67 index (%)	≤ 20	181 (34.8%)	116 (38.8%)	65 (29.4%)	0.044*
	(20, 50]	249 (47.9%)	139 (46.5%)	110 (49.8%)	
	> 50	90 (17.3%)	44 (14.7%)	46 (20.8%)	
NST outcome	pCR	201 (38.7%)	90 (30.1%)	111 (50.2%)	< 0.001*
	Non‐pCR	319 (61.3%)	209 (69.9%)	110 (49.8%)	
NST regimen	Chemotherapy	117 (22.5%)	71 (23.7%)	46 (20.8%)	0.429
	Chemotherapy and anti‐HER2 therapy	403 (77.5%)	228 (76.3%)	175 (79.2%)	
Reccurence	Suffered	102 (19.6%)	54 (18.1%)	48 (21.7%)	0.299
	None	418 (80.4%)	245 (81.9%)	173 (78.3%)	

Abbreviations: ER, estrogen receptor; HPBC, HR‐negative/HER2‐positive breast cancer; MLR, monocyte‐to‐lymphocyte ratio; NLR, neutrophil‐lymphocyte ratio; NST, neoadjuvant systemic therapy; pCR, pathological complete response; PR progesterone receptor; TPBC, triple‐positive breast cancer.

* *p* < 0.05 was considered statistically significant.

The TPBC and HPBC groups exhibited distinct clinicopathological profiles. A greater proportion of patients in the HPBC group had an elevated monocyte‐to‐lymphocyte ratio (MLR) (> 0.20) than those in the TPBC group (68.3% vs. 56.5%; *p* = 0.006), with additional significant differences in age (*p* < 0.001), menstrual status (*p* < 0.001), N stage (*p* = 0.002), histological grade (*p* = 0.048), and Ki67 index (*p* = 0.044). Consequently, the NST‐induced pCR rate was markedly lower in the TPBC group (30.1% vs. 50.2%; *p* < 0.001).

With respect to the neutrophil‐to‐lymphocyte ratio (NLR), sex hormone levels, tumor size, P53 status, and NST regimens, no significant intergroup differences were detected.

### 3.2 pCR as an Independent Predictor of Improved Survival in TPBC

**FIGURE 3:**
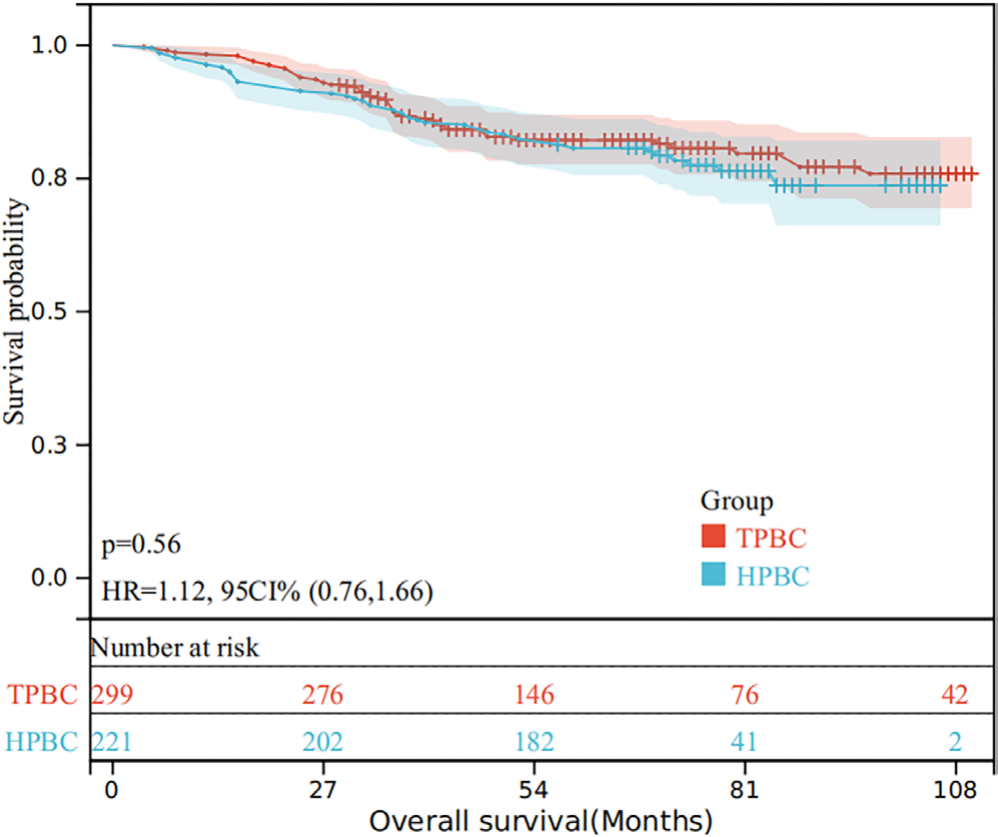
Kaplan–Meier analysis of DFS. Comparison of 5‐year DFS rates between the TPBC and HPBC patient groups. No statistically significant difference was observed (*p* = 0.56, 95% CI: 0.76–1.66).

Kaplan–Meier analysis was performed to compare 5‐year disease‐free survival (DFS) between the two cohorts. The 5‐year DFS rate was 82.8% in the TPBC group and 82.3% in the HPBC group, with no statistically significant difference (*p* > 0.05). The median follow‐up time was 72 months (Figure 3).

Although no significant difference in DFS was observed between the two groups, comparable long‐term outcomes did not necessarily imply identical predictors of short‐term treatment response. Given that TPBC represents a distinct molecular subtype with biomarker profiles differing from those of HPBC, we considered it essential to analyze treatment response and survival outcomes, particularly in TPBC patients.

In the TPBC cohort, univariate regression analysis for DFS revealed pCR as a significant prognostic factor (*p* = 0.012). Variables with *p* < 0.1 in the univariate analysis—namely age, ER status, P53 status, and pCR—were incorporated into a multivariate Cox model. Multivariate analysis confirmed that NST‐induced pCR was an independent predictor of improved 5‐year DFS (HR 0.340; 95% CI: 0.144–0.803; *p* = 0.014; Table 2).

**TABLE 2:** Univariate and multivariate regression analyses of 5‐year DFS in TPBC patients.

Characteristics	Univariate analysis HR (95% CI)	*p*	Multivariate analysis HR (95% CI)	*p*
Age				
> 45 vs. ≤ 45	0.592 (0.336–1.042)	0.069	2.168 (1.094–4.295)	0.072
Menopausal status				
Postmenopausal vs. Premenopausal	0.761 (0.413–1.401)	0.380		
NLR index				
> 1.96 vs. ≤ 1.96	1.025 (0.581–1.809)	0.932		
MLR index				
> 0.20 vs. ≤ 0.20	1.071 (0.605–1.895)	0.815		
E2				
> 61.5 vs. ≤ 61.5 pg/mL	1.002 (0.511–1.964)	0.995		
T staging				
T1	1 (reference)			
T2	0.781 (0.361–1.686)	0.528		
T3/T4	0.547 (0.190–1.575)	0.263		
N staging				
N0	1 (reference)			
N1	0.760 (0.372–1.552)	0.451		
N2/N3	1.086 (0.559–2.108)	0.808		
Histological grading				
III vs. I/II	0.850 (0.412–1.754)	0.660		
ER status (%)				
[1, 20)	1 (reference)		1 (reference)	
[20, 50)	2.146 (0.968–4.759)	0.060	1.975 (0.888–4.395)	0.095
≥ 50	1.092 (0.463–2.575)	0.841	0.996 (0.421–2.360)	0.993
PR status (%)				
≥ 50 vs. < 50	1.324 (0.746–2.351)	0.337		
Ki67 index (%)				
≤ 20	1 (reference)			
(20, 50]	1.149 (0.624–2.118)	0.655		
> 50	0.926 (0.368–2.334)	0.871		
P53 status				
Positive vs. negative	2.016 (0.944–4.307)	0.070	2.082 (0.974–4.451)	0.059
NST regimen				
Chemotherapy vs. chemotherapy and anti‐HER2 therapy	1.643 (0.769–3.512)	0.200		
NST outcome				
pCR vs. Non‐pCR	0.335 (0.143–0.789)	0.012*	0.340 (0.144–0.803)	0.014*

Abbreviations: CI, confidence interval; DFS, disease‐free survival period; ER, estrogen receptor; HR, hazard ratio; MLR, monocyte‐to‐lymphocyte ratio; NLR, neutrophil‐lymphocyte ratio; NST, neoadjuvant systemic therapy; pCR, pathological complete response; PR progesterone receptor; TPBC, triple‐positive breast cancer.

* *p* < 0.05 was considered statistically significant.

### 3.3 Construction of a Predictive Model for NST Efficacy in TPBC

Given that pCR was identified as the sole independent prognostic factor for 5‐year DFS in TPBC patients, we further evaluated the clinicopathological variables associated with pCR attainment following NST. The relationships between these factors and pCR are summarized in Table 3.

**TABLE 3:** Univariate and multivariate regression analyses of achieving pCR in TPBC patients.

Characteristics	Univariate analysis				Multivariate analysis			
	HR	5% CI	95% CI	*p*	HR	5% CI	95% CI	*p*
Age								
> 45 vs. ≤ 45	0.875	0.527	1.452	0.606				
Menopausal status								
Postmenopausal vs. premenopausal	0.751	0.446	1.267	0.283				
NLR index								
> 1.96 vs. ≤ 1.96	1.678	1.011	2.787	0.045*	1.327	0.726	2.425	0.358
MLR index								
> 0.20 vs. ≤ 0.20	1.835	1.096	3.072	0.021*	1.606	0.870	2.966	0.130
E2								
> 61.5 vs. ≤ 61.5 pg/ml	0.994	0.554	1.782	0.983				
T staging								
T1	1 (reference)							
T2	0.571	0.281	1.160	0.121				
T3/T4	0.447	0.182	1.097	0.079				
N staging								
N0	1 (reference)							
N1	0.764	0.425	1.375	0.370				
N2/N3	0.562	0.303	1.042	0.068				
Histological grading								
III vs. I/II	1.407	0.785	2.522	0.252				
ER status (%)								
[1, 20)	1 (reference)				1 (reference)			
[20, 50)	0.588	0.313	1.102	0.098	0.435	0.211	0.896	0.024*
≥ 50	0.451	0.239	0.849	0.014*	0.367	0.180	0.752	0.006*
PR status (%)								
≥ 50 vs. < 50	1.237	0.742	2.063	0.415				
Ki67 index (%)								
≤ 20	1 (reference)				1 (reference)			
(20, 50]	2.008	1.098	3.674	0.024*	2.179	1.141	4.164	0.018*
> 50	9.28	4.221	20.403	0.001*	12.038	5.054	28.674	0.001*
P53 status								
Positive vs. negative	1.201	0.686	2.101	0.522				
NST regimen								
Chemotherapy and anti‐HER2 therapy vs. Chemotherapy	3.297	1.602	6.787	0.001*	4.287	1.922	9.564	0.001*

Abbreviations: CI, confidence interval; ER, estrogen receptor; HR, hazard ratio; MLR, monocyte‐to‐lymphocyte ratio; NLR, neutrophil‐lymphocyte ratio; NST, neoadjuvant systemic therapy; pCR, pathological complete response; PR, progesterone receptor; TPBC, triple‐positive breast cancer.

* *p* < 0.05 was considered statistically significant.

Univariate analysis revealed that the NLR (*p* = 0.045), MLR (*p* = 0.021), NST regimen (*p* = 0.001), ER status (*p* = 0.014), and Ki67 index (*p* = 0.024) were significant predictors of pCR. Subsequent multivariate regression confirmed the NST regimen (*p* = 0.001), ER status (*p* = 0.024), and Ki67 index (*p* = 0.018) as independent factors for predicting pCR attainment following NST.

**FIGURE 4:**
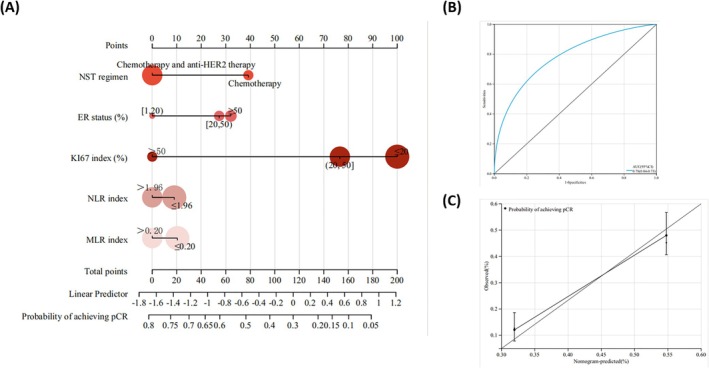
Predictive nomogram for NST response in TPBC. (A) Nomogram for estimating the probability of pCR. Each variable value corresponds to a specific score on the point scale; the sum of all scores is converted to a predicted pCR probability. (B) ROC curve of the nomogram, showing an AUC of 0.78 (95% CI: 0.73–0.83). (C) Calibration plot comparing the predicted probabilities with the observed pCR rates.

In summary, the NST regimen, ER status, NLR, MLR, and Ki67 index were identified as significant predictors of the NST response in TPBC patients. These five variables were subsequently integrated into a predictive nomogram to estimate the probability of achieving pCR (Figure 4A). According to the model, patients with high NLRs, high MLRs, low ER expression, and high Ki67 indices were predicted to have the highest likelihood of attaining pCR following combination therapy with anti‐HER2 agents and chemotherapy.

The predictive performance of the nomogram was evaluated by receiver operating characteristic (ROC) analysis, which revealed an area under the curve (AUC) of 0.78 (95% CI: 0.73–0.83). The model also showed a concordance index (C‐index) of 0.79. Furthermore, the calibration curves revealed good agreement between the predicted probabilities and observed outcomes, indicating moderate reliability of the prediction model (Figure 4B,C).

### 3.4 External Validation Confirms the Robust Predictive Performance of the Nomogram

**FIGURE 5:**
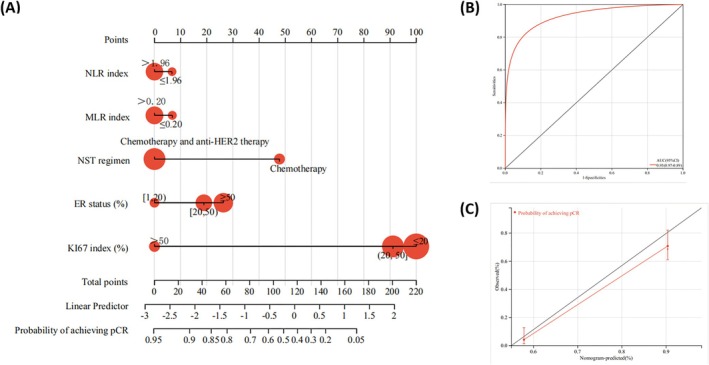
External validation of the predictive nomogram in an independent TPBC cohort (*n* = 143; January 2022–December 2024). (A) Application of the nomogram in the validation cohort. (B) ROC curve demonstrating robust discriminative ability (AUC = 0.93, 95% CI: 0.89–0.97). (C) Calibration plot showing agreement between the predicted probabilities and observed outcomes.

An external validation cohort comprising 143 TPBC patients diagnosed between January 2022 and December 2024 was used to assess the predictive ability of the nomogram. The validation results demonstrated strong consistency with those of the original model (Figure 5A). Specifically, the results of the ROC analysis revealed an AUC of 0.93 (95% CI: 0.89–0.97), with a C‐index of 0.77. Furthermore, the calibration curve indicated excellent consistency between the predicted and observed outcomes, confirming the model's robustness in the validation cohort (Figure 5B,C).

The high AUC observed in the external validation cohort may be attributable to more standardized diagnostic and therapeutic protocols implemented in recent years. During this period, nearly all TPBC patients received neoadjuvant dual anti‐HER2 therapy combined with chemotherapy, reflecting the contemporary standard of care. These findings further support the applicability of our model as a reference for future clinical decision‐making.

## 4 Discussion

NST improves the long‐term prognosis of breast cancer by downstaging primary tumors and metastatic lymph nodes. This downstaging effect can render initially inoperable lesions resectable, thereby expanding surgical options, increasing the rates of breast‐conserving surgery, and significantly enhancing patients' quality of life [9]. Numerous studies have shown that achieving pCR following NST significantly improves DFS and OS [13]. For patients who do not achieve pCR, the timely adjustment of postoperative adjuvant therapy is recommended [14]. Evidence further indicates that this association between pCR and survival benefit is particularly pronounced in TNBC and HER2‐positive breast cancer [15]. In our study, pCR was also an independent predictor of improved 5‐year DFS (HR 0.340; 95% CI: 0.144–0.803; *p* = 0.014) in TPBC patients.

The coexpression of HR and HER2 in TPBC introduces greater complexity in predicting the response to NST. Significant advances have been made in understanding the intracellular crosstalk between the ER and HER2 pathways, which promotes tumor progression and drug resistance through multilevel molecular interactions. Studies have revealed direct interactions between ERα and the HER2/EGFR pathway in the cytoplasm. Furthermore, ERα activation has been shown to selectively target tumor spheroid cells and confer resistance to trastuzumab [16]. This pathway crosstalk also sustains endocrine therapy resistance through cell cycle regulation [17]. Both ER signaling and HER2 signaling promote proliferation via cyclin D1 and CDK4/6 activation. In support of this mechanism, Gianni L et al. demonstrated that dual anti‐HER2 combined with the CDK4/6 inhibitor palbociclib significantly reduced Ki67 expression and improved pCR rates in TPBC [5]. Additionally, ER signaling is involved in nongenomic crosstalk through the activation of HER2 downstream effectors (MAPK/PI3K‐AKT), thereby compromising endocrine sensitivity [6]. Conversely, HER2 overexpression activates MAPK/mTOR signaling to phosphorylate ER or its coregulators (e.g., AIB1), maintaining ER transcriptional activity in a ligand‐independent manner and reducing tamoxifen efficacy [18]. ER activation also attenuates the anti‐HER2 response by upregulating survival proteins (e.g., BCL‐2) or enriching cancer stem cell populations [7]. Consequently, concurrent inhibition of both pathways or their shared downstream targets represents a promising therapeutic strategy for TPBC.

Growing clinical evidence confirms that intracellular crosstalk significantly influences the treatment response and prognosis in TPBC. Gianni et al. reported that the combination of docetaxel, trastuzumab, and pertuzumab resulted in a pCR rate of 45.8% in TPBC patients, while it resulted in a rate of 63.2% in the HR‐/HER2+ group [19]. In a multicenter phase II trial (NCT04486911), patients with newly diagnosed stage II–III TPBC received a triple oral NST regimen comprising pyrotinib, letrozole, and dalpiciclib. After five 4‐week cycles, the pCR rate reached 30.4% (95% CI: 21.3–41.3), accompanied by a significant reduction in the Ki67 index from 40.4% to 17.9% (*p* < 0.001) [20]. These findings collectively suggest that combining chemotherapy and anti‐HER2 agents with endocrine therapy can enhance treatment efficacy in TPBC.

Although long‐term survival did not significantly differ between the TPBC and HPBC groups in our study, the follow‐up period was ultimately limited. Furthermore, comparable survival outcomes do not equate to identical patterns of treatment response. In addition, the therapeutic behavior merits focused investigation on the basis of intracellular crosstalk between the ER and HER2 pathways in TPBC. In our study, a significant disparity was observed in the NST response, with the pCR rate being substantially lower in the TPBC group (30.1% vs. 50.2%, *p* < 0.001).

Multivariate analysis revealed that the NST regimen (*p* = 0.001), ER status (*p* = 0.006), and Ki67 index (*p* = 0.001) were independent factors influencing pCR attainment. In addition, a high NLR (*p* = 0.045) and MLR (*p* = 0.021) were also associated with pCR according to univariate analysis.

In our study, the combination of chemotherapy and anti‐HER2 therapy significantly increased the likelihood of achieving pCR (HR = 4.287; 95% CI: 1.922–9.564; *p* = 0.001). This finding is consistent with those of numerous studies indicating that anti‐HER2 therapy combined with chemotherapy improves both the pCR rate and survival rate in patients with HER2‐positive breast cancer. For instance, an EBCTCG meta‐analysis revealed that combination therapy reduced recurrence and mortality by approximately one‐third [21]. Furthermore, a multicenter retrospective study by Jiao D et al. suggested that patients with high HER2 expression benefited more from anti‐HER2 therapy, whereas those with low HER2 expression or HR‐positive status exhibited relatively poor responses [22]. These results underscore the importance of tailoring combination therapy based on individual tumor characteristics, such as HER2 expression level and HR status.

Lower ER expression was significantly associated with improved treatment response in our cohort (HR = 0.435; 95% CI: 0.211–0.896; *p* = 0.024; HR = 0.367; 95% CI: 0.180–0.752; *p* = 0.006). Consistent with this, the pCR rate in TPBC was notably lower than that in the other HPBC subtypes. Chen H et al. reported similar findings in a real‐world study in which the pCR rates were significantly lower in HR^+^/HER2^+^(only ER+ or PR+) and TPBC groups than in the HR^−^/HER2^+^ group (24.3% and 36.9% vs. 49.2%, *p* < 0.001) [23]. We propose that this discrepancy may be attributed to intracellular crosstalk between the ER and HER2 pathways. Furthermore, Bruss C et al. demonstrated that ER‐positive breast cancer cells can modulate the tumor microenvironment, thereby reducing responsiveness to immunotherapies such as PD‐1 inhibitors [24].

A high Ki67 index is widely recognized as a biomarker of tumor proliferation activity and is predictive of an enhanced response to antitumor therapy. In our analysis, elevated Ki67 levels were significantly associated with increased likelihood of achieving pCR (HR = 2.179; 95% CI: 1.141–4.164; *p* = 0.018; HR = 12.038; 95% CI: 5.054–28.674; *p* = 0.001). These findings align with those of the NCT04486911 trial, which reported promising pCR rates following treatment with pyrotinib, letrozole, and dalpiciclib in TPBC patients with high baseline Ki67 expression [20]. Moreover, dynamic monitoring of Ki67 during NST may enable early efficacy assessment and guide treatment adaptation. For instance, the NA‐PHER2 trial demonstrated a significant decrease in Ki67 and a 27% pCR rate in TPBC patients treated with dual anti‐HER2 therapy and palbociclib [5]. In addition, Martins‐Branco D et al. reported that residual Ki67 expression in postoperative specimens correlated with long‐term DFS, underscoring its prognostic relevance [25].

Univariate regression analysis revealed significant associations between both the NLR and the MLR and pCR (NLR: HR = 1.678; 95% CI: 1.011–2.787; *p* = 0.045; MLR: HR = 1.835; 95% CI: 1.096–3.072; *p* = 0.021). As systemic inflammatory markers, the NLR and MLR may influence the treatment response by modulating the inflammatory tumor microenvironment. Nevertheless, their predictive value for NST remains incompletely understood. Campbell M J et al. reported that an elevated NLR was related to attenuated immune activation following chemotherapy, potentially contributing to reduced pCR rates [26]. Wang X et al. proposed that dynamic changes in the MLR during treatment could serve as early efficacy indicators, although further validation is warranted. In their study, the systemic inflammatory response index, a composite indicator integrating neutrophil and MLR, was identified as an independent predictor of the efficacy of NST [27]. Moreover, the predictive performance of these inflammatory markers may be substantially influenced by specific treatment modalities, including the use of anti‐HER2 agents or immune checkpoint inhibitors [26].

In summary, we developed a clinically applicable nomogram to predict the probability of achieving NST‐induced pCR in TPBC patients. The model revealed that TPBC patients with high NLRs, high MLRs, low ER expression, and high Ki67 indices were more likely to achieve pCR. These findings suggested that the nomogram could assist in stratifying patient risk and optimizing treatment strategies, particularly for those who are predicted to have a poor response.

Current NSTs for TPBC focus on dual anti‐HER2 therapy combined with chemotherapy. However, the efficacy of this approach is often limited by intracellular crosstalk between the ER and HER2 signaling pathways. Emerging therapeutic strategies, particularly CDK4/6 inhibitors in combination with endocrine and anti‐HER2 agents, show considerable promise. For instance, the MUKDEN 01 trial demonstrated that a regimen containing pyrotinib and dalpiciclib achieved a pCR rate of 30.4%, highlighting the potential of targeted combination therapies in this setting [20]. Recent advances highlight the evolving therapeutic landscape for TPBC. At the 2024 San Antonio Breast Cancer Symposium, updated results from the PATINA trial demonstrated that adding the CDK4/6 inhibitor palbociclib to anti‐HER2 therapy and endocrine therapy significantly extended DFS by more than 15 months (HR = 0.74; 95% CI: 0.58–0.94; *p* = 0.007) in advanced TPBC. Beyond CDK4/6 inhibition, T‐DXd (trastuzumab deruxtecan) has shown efficacy in HER2‐low breast cancer in DESTINY‐Breast trials, suggesting potential future applications in TPBC [28]. Genomically, TP53 mutations are highly prevalent in TPBC, and targeted therapies are under investigation [29]. Preclinical studies also indicate that combined inhibition of the PI3K/AKT and Wnt pathways may overcome treatment resistance, although clinical validation is needed [30]. Furthermore, the KATHERINE trial established T‐DM1 as a standard adjuvant therapy for patients who did not achieve pCR following NST, including those with TPBC [31]. These developments reflect a shift from conventional chemotherapy and anti‐HER2 regimens toward multitarget and immune‐modulatory strategies, warranting further clinical evaluation.

This nomogram was developed to inform real‐world clinical decision‐making in TPBC patients. For patients identified as high risk for poor prognosis by the model, intensified preoperative strategies may be considered—such as incorporating novel oral endocrine agents during NST or early enrollment in clinical trials of investigational drugs. These individuals also warrant closer surveillance. If a suboptimal response persists, earlier planning for more extensive radical surgery should be considered. Conversely, for patients who are predicted to have a high likelihood of achieving pCR, the potential for treatment de‐escalation—including the avoidance of highly toxic chemotherapy agents—merits further investigation. While the individualized predictions provided by this model may serve as a valuable supplement to standard treatment decisions, prospective validation through large‐scale multicenter trials and further refinement are warranted before broad clinical implementation.

Our study has several limitations inherent to its single‐center, retrospective design, including potential information and selection biases. Given that all the data were derived from a single institution, our findings may be limited by regional or institutional specificities. Future work will incorporate multicenter data to expand the study population and examine potential ethnic and geographic variations in the NST response among TPBC patients.

Obtaining robust long‐term survival data was not feasible in the current study because of the finite follow‐up period. Accordingly, we prioritized pCR as the primary endpoint, recognizing that a well‐validated pCR prediction model can itself serve as a valuable clinical decision‐support tool in routine practice. Nevertheless, continued follow‐up of this cohort is underway, and future efforts will aim to develop predictive models specifically for long‐term survival outcomes in TPBC patients. Additionally, the statistical power of our analysis was constrained by the sample size, underscoring the need for larger, prospective cohorts.

Notably, the enrollment period of this study coincided with a transformative era in the standard of care for HER2‐positive breast cancer. Prior to 2018, a subset of patients diagnosed at our institution did not receive anti‐HER2 targeted therapy during the neoadjuvant phase because of insurance restrictions or financial constraints. These patients predominantly underwent neoadjuvant chemotherapy alone, followed by standard adjuvant anti‐HER2 therapy after surgery. From 2019 onward, with the regulatory approval and subsequent reimbursement of pertuzumab for neoadjuvant use in China, dual anti‐HER2 blockade combined with chemotherapy became widely adopted at our center. Consequently, most patients treated after this time point received concurrent neoadjuvant chemotherapy and anti‐HER2 targeted therapy. We acknowledge that these factors may influence treatment response and confound outcome interpretation. Nevertheless, the NST regimen was confirmed as an independent predictor of pCR by both univariate and multivariate analyses. Importantly, no significant intergroup difference in the NST regimen distribution was observed, supporting the validity of our findings regarding the predictive value of treatment response and survival outcomes. Ongoing efforts are directed toward continued data collection from TPBC patients under current standardized protocols, aiming to refine our predictive model and further validate its clinical utility.

## 5 Conclusion

The management of TPBC remains challenging because of its inherent resistance to conventional therapies and the scarcity of subtype‐specific targets. To address this, we developed a predictive model for the NST response in TPBC, aiming to stratify early risk. For those classified as high risk by the model, we recommend implementing biomarker‐guided NST strategies and intensifying follow‐up surveillance to optimize treatment efficacy and disease control. For patients who are predicted to have a high likelihood of achieving pCR, treatment de‐escalation may be considered to mitigate drug‐related toxicity and reduce therapeutic burden.

## Funding

This work was supported by the Clinical Medicine First‐Class Discipline Construction Project (CYYY‐BSYJSCXXW‐202322).

## Ethics Statement

This observational study was conducted in accordance with the Declaration of Helsinki and was approved by the Ethics Committee of the First Affiliated Hospital of Chongqing Medical University (April 29, 2020/No. 2022‐202). The requirement for informed consent was waived by the committee.

## Conflicts of Interest

The authors declare no conflicts of interest.

## Data Availability

The datasets generated and analyzed during this study were publicly available in the Dryad repository: https://datadryad.org/dashboard (DOI: https://doi.org/10.5061/dryad.wpzgmsc2g).
